# Mining frequent patterns for AMP-activated protein kinase regulation on skeletal muscle

**DOI:** 10.1186/1471-2105-7-394

**Published:** 2006-08-30

**Authors:** Qingfeng Chen, Yi-Ping Phoebe Chen

**Affiliations:** 1 School of Engineering & Information Technology, Deakin University, Melbourne, Australia; 2 Australia Research Council (ARC) Centre in Bioinformatics, Australia

## Abstract

**Background:**

AMP-activated protein kinase (AMPK) has emerged as a significant signaling intermediary that regulates metabolisms in response to energy demand and supply. An investigation into the degree of activation and deactivation of AMPK subunits under exercise can provide valuable data for understanding AMPK. In particular, the effect of AMPK on muscle cellular energy status makes this protein a promising pharmacological target for disease treatment. As more AMPK regulation data are accumulated, data mining techniques can play an important role in identifying frequent patterns in the data. Association rule mining, which is commonly used in market basket analysis, can be applied to AMPK regulation.

**Results:**

This paper proposes a framework that can identify the potential correlation, either between the state of isoforms of α, β and γ subunits of AMPK, or between stimulus factors and the state of isoforms. Our approach is to apply item constraints in the closed interpretation to the itemset generation so that a threshold is specified in terms of the amount of results, rather than a fixed threshold value for all itemsets of all sizes. The derived rules from experiments are roughly analyzed. It is found that most of the extracted association rules have biological meaning and some of them were previously unknown. They indicate direction for further research.

**Conclusion:**

Our findings indicate that AMPK has a great impact on most metabolic actions that are related to energy demand and supply. Those actions are adjusted via its subunit isoforms under specific physical training. Thus, there are strong co-relationships between AMPK subunit isoforms and exercises. Furthermore, the subunit isoforms are correlated with each other in some cases. The methods developed here could be used when predicting these essential relationships and enable an understanding of the functions and metabolic pathways regarding AMPK.

## Background

In recent years, there has been a tremendous growth in biological data and the emergence of new, efficient experimental techniques. A variety of genomic and proteomic databases are now publicly accessible over the Internet. However, it is widely recognized that the mere gathering of discrete data is insufficient for us to discover the potential correlations amongst them. The biological interpretation and analysis of these data are crucial. Such biological data not only provides us with a good opportunity for understanding living organisms, but also poses new challenges. This has led us to the development of a new method to analyze the data.

**Figure 1:**
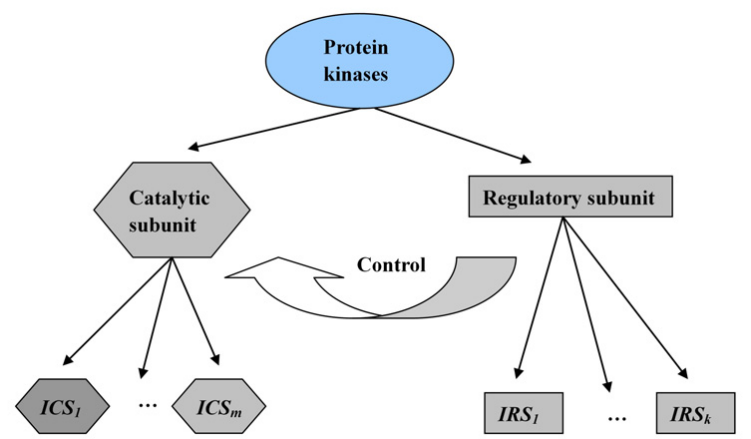
Catalytic subunit and Regulatory subunit of Protein kinases, in which *ICS *and *IRS *represent the isoform of catalytic subunit and regulatory subunit respectively.

Protein kinases' regulation data can be a good foundation for understanding their structure, function, and expression. One goal, in terms of analyzing protein kinase regulation data, is to determine how an external stimulus might affect the catalytic subunit and regulatory subunit of protein kinases. Figure 1 presents Protein kinase *X *uses *IRS*_*i*_, a regulatory subunit (a second protein molecule) to control the activity of a catalytic subunit *ICS*_*j*_. Each subunit consists of several gene encoding isoforms. Another goal is to determine what isoforms are expressed or unchanged in expression as a result of certain conditions. AMP-activated protein kinase (AMPK) has recently emerged as a potential key signalling pathway, in the regulation of exercise-induced changes in glucose and lipid metabolism in skeletal muscle [1,2]. This enzyme is activated as a result of the alterations in cellular energy levels [3]. The activation of AMPK also exerts long-term effects at the level of both gene expression and protein synthesis, such as positive effects on glucose uptake of heart, food intake of hypothalamus, and negative effects on insulin secretion of pancreas and cholesterol synthesis of liver [4]. Hence, the investigation into the degree of activation and deactivation of subunit isoforms of AMPK will contribute to a greater understanding of AMPK and disease treatment [5].

Unfortunately, the traditional computational methods have been mainly used in sequence alignment [6], gene prediction [7], and microarray analysis. However, the efforts to develop robust methods to analyze AMPK regulation data lag behind the rate of data accumulation. Most of the current analysis rests on isolated discussion of single experimental results. Also, there has been a lack of systematical collection and interpretation of diverse AMPK regulation data. Besides, the existing approaches that seek to analyze biological data cannot cope with the AMPK regulation data that contains status messages of subunit isoforms and stimulus factors. This calls for the use of sophisticated computational techniques.

Recently, data mining techniques have emerged as a means of identifying patterns and trends from large quantities of data. Among them, association rule mining is a popular summarization and pattern extraction algorithm to identify correlations between items in transactional databases [8]. Several attempts have been made to mine biological databases using association rule mining [9-11]. Earlier investigations mainly focus on discovering an association between the gene expression, genetic pathways and protein-protein interaction. However, not much work has been found to address AMPK regulation data. Hence, it is necessary to identify implicit, but potentially useful, frequent patterns from the AMPK regulation data.

An association rule is an implication of the form *X *⇒ *Y*, where *X *and *Y *are itemsets. For example, as for AMPK regulation, *Y *and *X *can represent the subunit isoforms of AMPK that is highly expressed or unchanged in expression, and stimulus factors that describe the certain conditions such as the intensity and duration, respectively. A rule might be {moderate intensity} ⇒ {α_1a_|, α_2a_↑} where α_1a _and α_2a _represent the activity of α subunits of AMPK. It shows that α_1a _usually has no change in expression, and α_2a _is highly expressed in most experiments where the exercise intensity is moderate. Typically, this method requires a user to specify a minimum support threshold for the generation of all itemsets. However, without specific knowledge, it is difficult for users to set the support threshold. Therefore, it would be better to identify top-*N *interesting itemsets, instead of specifying a fixed threshold value for all itemsets of all sizes like [12].

This paper presents a framework by which to analyze the AMPK regulation data derived from the published experimental results. FPtree (Frequent Pattern Tree) algorithm [13] is used to extract frequent itemsets efficiently, and item constraints in the closed interpretation are proposed to specify general constraints on itemset generation. A number of rules of interest are discovered from mining AMPK data. A cursory analysis of some of the like reveals numerous potential associations between the states of subunit isoforms of AMPK, or between the stimulus factor and the state of isoforms, many of which make sense biologically. Those suggesting new hypotheses may warrant further investigation. If a data set from existing experiments has missing values for some subunit isoforms that are intentionally untested in corresponding experiments, these items are filtered out ahead of mining. Furthermore, the items that are not tested by adequate experiments will be reserved in databases for future use.

This paper is organised as follows. In section 'Methods', the basic concepts used in this paper, are discussed. Section 'Mining AMPK' with item constraints, presents the procedure to find association rules from AMPK regulation data. Section 'Results' explains implementation of the algorithm to discover association rules using experiments. Section 'Discussion' refers to our methodology and future directions. Finally, this paper is concluded in section 'Conclusions'.

## Methods

### The basics of association rule mining

An association rule is an implication of the form, *A *⇒ *B*, where *A *and *B *are itemsets, and *A *∩ *B *= ∅. The following criteria can be used to evaluate the association rule:

1. *support *for a rule *A *⇒ *B *is the percentage of transactions in *D *that contain *A *∪ *B*, and is defined as *supp*(*A *∪ *B*); and

2. *confidence *for a rule *A *⇒ *B *is defined as *conf*(*A *⇒ *B*) = *supp*(*A *∪ *B*)/*supp*(*A*).

According to the support-confidence framework [14], a rule *A *⇒ *B *is of interest if *supp*(*A *∪ *B*) ≥ *minsupp *and *conf *(*A *⇒ *B*) ≥ *minconf*. In this article, the conventional association rule mining is extended for analyzing AMPK regulation data. Suppose *E *= {*E*_1_, ..., *E*_n_} is a set of experiments. Each experiment consists of an *eid *(experiment identifier) and two itemsets, *E*_*i *_= (*eid*, *S*_i_, *ST*_i_); *ST*_i _⇒ *S*_i _is treated as an initial rule. Let *I *= {*x *| *x *∈ *S*_i _∪ *ST*_i_, 1 ≤ *i *≤ n} be a set of items, and *A *⊆ *I *and *B *⊆ *I *be itemsets. A rule *A *⇒ *B *has support, *s*, in the set of experiments if *s*% of experiments contains *A *and *B*. The rule has confidence, *c*, if *c*% of experiments containing *A*, also contain *A *and *B*.

### Definition of problem

For regulating critical biological processes such as memory, hormone responses, and cell growth, living organisms rely on a family of enzymes called *protein kinases*. In particular, AMPK is activated in skeletal muscle in response to exercise, phosphorylating target proteins along diverse metabolic pathways, such as glucose uptake and fatty-acid oxidation. The response to environmental demands is accomplished by signal transduction by which an extracellular signal interacts with receptors at the cell surface, activating factors in signaling pathways and ultimately sustaining muscle performance by activating skeletal muscle remodeling genes. Furthermore, recent findings point to the AMPK pathway as a potential target for therapeutic strategies to restore metabolic balance to patients, such as type 2 diabetic and obesity patients. Thus, AMPK pathway is a particularly challenging problem in bioinformatics.

**Figure 2:**
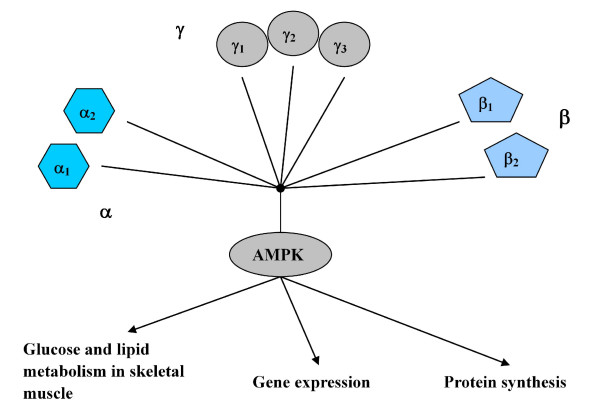
Subunit isoforms of AMPK and its functions.

Recent studies [1,2] have shown that AMP-activated protein kinase (AMPK) plays an important role in the regulation of exercise-induced changes, which occur in glucose and lipid metabolism in skeletal muscle, and gene expression and protein synthesis. AMPK contains *catalytic subunit isoforms *α_1 _and α_2 _and *regulatory subunit isoforms *β_1_, β_2_, γ_1_, γ_2 _and γ_3_, and the regulation controlled by subunits is shown in Figure 2. The subunit isoforms congregate together to perform the functions of AMPK. Therefore, interpretation and analysis of AMPK regulation data and identification of potential associations from the data, may lead to a better understanding of its structure, function and expression. However, only limited studies have been conducted to analyze this valuable data, and the majority of these studies have focused on isolated experiments. In this article, a framework is proposed to discover association rules from a collection of AMPK regulation data.

Given a set of experimental results, *E*, each result is described by a set of items. The items can be classified into two categories:

1. *Stimulus item *(*ST*) represents a parameter that is used to measure stimuli. For example, intensity, load and duration are generally used to measure the exercise stimuli. +++, ++, + and - indicate that the stimulus is "*intense*", "*high*", "*moderate*" and "*low*", respectively.

2. *State item *(*S*) represents isoforms of α, β and γ subunits of AMPK. ↑, | and ↓ are used to indicate "*highly expressed*", "*expressed*" and "*no change*" in expression, respectively.

Items collected from different experiments contain a great deal of hidden information that may be meaningful. For example, in an observed experiment based on the training of moderate intensity treadmill, β_2 _and γ_2 _isoforms of AMPK are found to be highly expressed in terms of activation in white quadriceps [15]. The initial rule for this experiment can be written as *A *= {*moderate intensity treadmill*} ⇒ *B *= {β_2a_↑, γ_2a_↑}. It is hypothetically derived from an experiment. Nevertheless, in practice, the association rules have to be mined from an experimental data set.

The best known strategy to mining frequent itemsets is Apriori [14], which lives on the essential assumption that all itemsets have a uniform minimum support. However, in reality, the minimum support is not uniform. For example, rules containing *coffee *and *milk *or *coffee *and *sugar *usually have higher support than rules containing *coffee *and *tea*. The occurrence of a large itemset is inherently smaller than that of a small itemset in accordance with probability [16]. On the other hand, it is still troublesome for users to specify an optimal support threshold for all itemsets of all sizes. If the threshold is set too small, there may be too many results for the users. This may be time consuming during the computational phase and result in extra efforts to sort out answers of interest. If it is too large, there may be only a small number of results. In that case, some useful results can be missed. Thus, the constraint-based mining technique has been highlighted in recent work [12]; it provides a flexible way for users to specify a set of constraints and allow them to search and control the interesting frequent patterns. For example, in AMPK regulation, we may only want to know the relationships among items of different attributes, such as the activity of α_1_*isoform *and *stimulus factors*, e.g. intensity and duration. Their explanation will be described in the next two sections.

## Mining AMPK regulation with item constraints

### Deriving initial items

In general, mining of association rules starts with generating all frequent itemsets. The mined data set can be derived from different experimental results. The experimental conditions have to be considered in order to find out correct association rules. To discover frequent itemsets, initial items from experimental data, including experimental conditions such as moderate intensity and treadmill training, are to be derived. Also, the item derived from AMPK regulation data includes not only its status measurement but also item name such as α_1a _| and β_2a_↑.

In AMPK regulation data, *activity*, *protein expression *and *phosphorylation *are used as testing indexes for α, whereas only *protein expression *is used for β and γ. The following steps can be used to generate initial items from experimental data:

- Generate initial rules *ST *⇒ *S *from experimental data where *S *and *ST *represent *state itemset *and *stimulus itemset*, respectively and

- Derive a set of initial items *I *= *S *∪ *ST*.

It is illustrated in the form of {*x*_1_, *x*_2_, ..., *x*_n_} where *x*_i _∈ *I*. Let the experimental universe be *EID *= {*E*_1_, *E*_2_, *E*_3_, *E*_4_}. Table 1 represents an example of initial items derived from different experiments. Each row in Table 1 corresponds to an experiment for AMPK regulation. "-" represents that this item is untested in the corresponding experiments. There are four initial rules, namely, 1) {φ^+^, φ^-^} ⇒ {α_1a_↓, α_1p_↑}, 2) {φ^+^, φ^-^} ⇒ {α_1a _↓, α_1e_|, α_1p_|, β_1e_|, β_2e_|}, 3) {φ^+^, φ^+^} ⇒ {α_1a_↓, α_1e_|, β_1e_|} and 4) {φ^++^, φ^+^} ⇒ {α_1a_|, α_1e_↓, α_1p_|, β_1e_|, β_2e_|} where subscripts *a*, *p *and *e *represent *activity*, *phosphorylation *and *protein expression*, respectively. Therefore, ∀ *A *⊆ {α_1a_↓, α_1a_|, α_1e_|, α_1e_↓, α_1p_↑, α_1p_|, β_1e_|, β_2e_|, φ^+^, φ^++^, φ^-^, φ^+^} is an itemset and ∀ *y *∈ {α_1a_↓, α_1a_|, α_1e_|, α_1e_↓, α_1p_↑, α_1p_|, β_1e_|, β_2e_|, φ^+^, φ^++^, φ^-^, φ^+^} can be treated as an initial item.

In order to determine association rules, the frequent itemsets need to be sorted out from the obtained initial items.

### Specifying item constraints

*FP-tree *algorithm [13] constructs a frequent pattern tree that has an extended prefix-tree structure and stores quantitative information about frequent patterns. It can generate a complete set of frequent patterns. The typical data format for using this algorithm is shown in Table 1. We notice that not every item does occur in all experiments in this Table. Our method marks these items with *filtering symbols *so as to filter out the itemsets that contain such items during the itemset generation. Actually, if an itemset is not tested in most of the experiments, it will not be considered when finding association rules for the time being, instead it will be reserved for future use.

Suppose *support*(*X*, *E*) denotes the support of *X *in experiment *E *and *f*(*X*, *E*) represents the occurrence of itemset *X *in *E*, it can be defined as:

*support*(*X*, *E*) = |*f*(*X*, *E*)|/|*E*|     (1)

where *f*(*X*, *E*) = {*E*_*i *_∈ *E *| *E*_*i *_contains *X*}. For example, *support*(α_1e_|, *E*) in Table 1 is 2/4 or 50%.

*X *is a high occurrence itemset if *support*(*X*, *E*) ≥ *minoccur *(*minimum occurrence*). To avoid missing valuable initial itemsets, we assume *minoccur *= 2/|*E*|, which is the lower bound of minimum support corresponding to the support requirement of at least 2 experiments. Otherwise, it is called a *low occurrence itemset*. Consequently, a collection of *initial interesting itemsets *can be obtained in light of the initialized *filtering symbols *and given *minimum occurrence*.

To extract frequent itemsets, users usually need to specify a fixed minimum support but this method has been criticized due to its difficulty [12,13,16]. As described above, it is quite subtle to set a minimum support: a too small threshold may result in the output of many redundant patterns, whereas a too big one may generate no answer or miss interesting knowledge. In addition, the probability of occurrence of a larger size itemset is inherently much smaller than that of a smaller size itemset [12]. A more flexible option is to allow users to specify different thresholds in accordance with different itemsets [12]. Consequently, the *top-N *interesting itemsets are returned as answers.

Suppose a ***k*-*itemset ***denotes a set of items containing *k *items. We have the following two definitions.

**Definition 1**. ***Top*-*N *interesting ***k***-itemsets**. Suppose a ***k*-itemset **is sorted in descending order by their supports. Let *s *be the support of the *Nth k-itemset *in the sorted list. The ***top*-*N *interesting ***k***-itemsets **represent the set of *k*-*itemsets *whose supports are equal to or larger than *s*.

From the observation, it is possible that there are multiple itemsets that have the same support *s*. The ***top*-*N *interesting ***k***-itemsets **may contain more than *N *itemsets. In this extreme case, it will be reported to the user, rather than returning all of them [12].

**Definition 2**. ***Top*-*N *interesting itemsets **is the union of the ***top-N *interesting *k*-itemsets **where 1 ≤ *k *≤ *k*_*max *_and *k*_*max *_is the upper bound of the size of itemsets we would like to find. An itemset is of interest if, and only if, it is in the ***top*-*N *interesting itemsets**.

EXAMPLE 3.1. In Table 1, we specify *k*_*max *_= 7 because the maximal tested items in every experiment are 7. The **interesting *1*-itemsets **is {α_1a_↓, α_1e_|, α_1p_|, β_1e_|, β_2e_|, φ^+^, φ^-^, φ^+^} after filtering α_1a_|, α_1e_↓, α_1p_↑ and φ^++ ^by formula (1). As a result, ***top-1 *interesting 1-itemsets **= {α_1a_↓} and ***top-2 *interesting 1-itemsets **= {α_1a_↓, β_1e_|, φ^+^}.

From the observation, it is clearly impractical to enumerate all ***top*-*N *interesting itemsets**. Although several ***top-N ***mining algorithms [12,13,16,17] already exist, they focus, without exception, on finding all ***top*-*N *itemsets**. However, this may be time-consuming and can lead to many useless or uninteresting itemsets. For example, itemsets from {*training*, *glycogen*, *diabetes*, *nicotinic acid*, *intensity*, *duration*} rather than their subsets are of interest because a part of the testing indexes cannot correctly describe an experiment. Our method is to partition a set of items, *I*, into several bins, where each bin *B*_*j *_contains a subset of items in *I*. We use item constraint in a similar way to the enumeration-based specification defined in [16] so that the items in a bin are not distinguished in terms of the specification. For example, *B*_1 _= {β_1e_, β_2e_} and *B*_2 _= {α_1a_, α_1e_} represent that the user is interested in protein expression of β, rather than α and γ, and only *activity *and *protein expression *of α, rather than *phosphorylation*. The constraint can be expressed in the following brief formula:

*IC*_*i*_(*B*_*i*1_,…, *B*_*im*_) = *N*_*i *_    (2)

where *B*_*ij *_∩ *B*_*ik *_= ∅, 1 ≤ *j*, *k *≤ *m*, *j *≠ *k*. *N*_i _is the number of itemsets satisfying *IC*_*i *_in terms of a derived support described in Section 'Results'. It explicitly defines what particular items we focus on and which should be presented in the identification of frequent patterns.

The concept of **closed interpretation **in [16] is adopted as well. An itemset *X *∈ *I *satisfies a constraint *IC*_*i *_in the closed interpretation if *X *contains exactly one item from each *B*_*ij *_in *IC*_*i*_, and these items are completely different. Suppose *IC*_*j *_is an item constraint, and |*IC*_*j *_| = *k*. As for close interpretation, a collection of *k*-*itemsets *can be generated. These itemsets are sorted in light of their support in descending order. Let the *N*_*j*_th greatest support be θ. Consequently, all *k*-*itemsets*, with support not less than θ, are interesting due to the constraint. We call them **top-*N*_*j *_interesting itemsets **of *IC*_*j *_in the closed interpretation. Usually, users can specify several item constraints. In this scenario, we want to find **top-*N*_*i *_interesting itemsets **for each *IC*_*i *_in the closed interpretation.

Suppose, for example, we partition the items in Table 1 into seven bins: *B*_1 _= {α_1a_↓, α_1a_|}, *B*_2 _= {α_1e_|, α_1e_↓}, *B*_3 _= {α_1p_↑, α_1p_|}, *B*_4 _= {β_1e_|}, *B*_5 _= {β_2e_|}, *B*_6 _= {φ^+^, φ^++^}, *B*_7 _= {φ^-^, φ^+^. Let *IC*_1 _(*B*_1_, *B*_2_, *B*_3_) = 2, *IC*_2 _(*B*_4_, *B*_5_) = 3 and *IC*_3 _(*B*_6_, *B*_7_) = 2. Consider itemset *X*_1 _= {α_1a_↓, α_1e_|}, *X*_2 _= {β_1e_|, β_2e_|} and *X*_3 _= {φ^+^, φ^-^}, they correspond to bin patterns (*B*_1_, *B*_2_, *B*_3_), (*B*_4_, *B*_5_) and (*B*_6_, *B*_7_), respectively. Therefore, we say that *X*_1 _satisfies *IC*_1 _in the closed interpretation;*X*_2 _satisfies *IC*_2 _in the closed interpretation; and *X*_3 _satisfies *IC*_3 _in the closed interpretation.

Until now, we have not referred closely to how the filtering symbols are specified. The untested items in experiments need to be pruned before going on to set up item constraints. Our approach converts the initial items of experiments into the format of non-negative integer. Consequently, each bin can contain several non-negative integers but any two bins must be disjoint. Table 2 is a conversion of Table 1, in which 8, 12, 29 and 30 correspond to the untested items regarding α_1e_, α_1p_, β_1e _and β_2e_, respectively. Therefore, the bins defined in the last paragraph can be converted into *B*_1 _= {1, 2}, *B*_2 _= {5, 6}, *B*_3 _= {10, 11}, *B*_4 _= {26}, *B*_5 _= {29}, *B*_6 _= {91, 92}, and *B*_7 _= {100, 101}. These filtering symbols are reserved in the data set and will be skipped when finding patterns from the *initial interesting itemsets*.

EXAMPLE 3.2. In Table 2, the non-negative integers 8, 12, 27 and 30 represent the specified filtering symbols. They correspond to the untested items with respect to α_1e_, α_1p_, β_1e _and β_2e_, respectively.

### Discovering association rules

The obtained **top-*N*_*i *_interesting itemsets **for item constraints in the closed interpretation can then be used to construct association rules. There are two approaches to identify association rules, depending on which antecedents or consequents of rules the item constraint *IC *are defined. Let *X *and *X' *be top interesting itemsets from *IC *and *IC'*, respectively, and *X' *⊂ *X*.

1. if *supp*(*X*)/*supp*(*X*^'^) ≥ *minconf*, *X' *→ *X *- *X' *is a rule of interest.

2. if *supp*(*X*)/*supp*(*X *- *X'*) ≥ *minconf*, *X *- *X' *→ *X' *is a rule of interest.

In addition, those rules that do not make sense biologically are pruned while mining AMPK regulation data. Hence, the valid rules should conform to the rules as given below:

- *Rule1*: *A *⇒ *B*, *A *⊆ *S *and *B *⊆ *S *and

- *Rule2*: *A *⇒ *B*, *A *⊆ *ST *and *B *⊆ *S*.

If the antecedent or consequent of a rule contains either both state items and stimulus items, or only stimulus items, it will be pruned. Obviously, the relation between only stimulus items such as *intensity *and *time *is not meaningful in biology at all. Besides, state items actually rely on stimulus items; therefore, they should not be included in the antecedent and consequent of a rule simultaneously. These kinds of rules are uninteresting and need to be pruned, and the search space can be reduced.

## Results

### Pre-processing of experimental data

FP-tree algorithm is applied to generate all frequent itemsets and find association rules that make sense biologically. We experimented on the published experimental data of AMPK regulation, in which AMPK is activated by endurance training in human skeletal muscle. The data is collected through searching the NCBI database and surely ranges over all the openly available data. Originally, the data contained 17 attributes, 46 items and 45 experiments [see Additional file 1]. After conversion into the format of non-negative integer, there are 57 items owing to the insert of filtering items [18]. In addition, initial items from experiments are divided into state items and stimulus items. Each item corresponds to an attribute/value pair.

AMPK consists of state items:*catalytic subunit *(α) and *regulatory subunit *(β, γ). The isoforms (α_1_, α_2_) of α are measured by *activity*, *phosphorylation *and *protein expression *but the isoforms of β and γ (β_1_, β_2_, γ_1_, γ_2_, γ_3_) are measured only by *protein expression*. As a result, there are 11 state items including all isoforms of AMPK, α_1a_, α_1e_, α_1p_, α_2a_, α_2e_, α_2p_, β_1e_, β_2e_, γ_1e_, γ_2e _and γ_3e_. Six stimulus indexes, including *training*, *glycogen*, *diabetes*, *nicotinic acid*, *intensity *and *duration*, are considered.

Table 3 is a random example of the activity and expression of AMPK in skeletal muscle, in which *N*, *I *and *D *represent *nicotinic acid*, *intensity *and *duration*, respectively. Each column corresponds to an experiment. The subscript *a *and *e *means *activity*, and *protein expression*, respectively. 1 and 5 represent *no change *in corresponding items; 2 and 6 represent *expressed*; 50 and 51 represent *trained *and *untrained*, respectively; 60 represents *normal glycogen*; 70 represents *normal nicotinic acid*; 100, 101 and 103 represent the duration under 20 minutes, between 20 and 60 minutes and above 90 minutes, respectively; 90, 91 and 92 represent the *maximal oxygen uptake *(VO(2)max) under 50%, VO(2)max between 65% and 75%, and VO(2)max between 80% and 100%, respectively.

To generate constraint specification, we group the items from the same attribute into a bin, yielding 17 bins for the 17 attributes. Although each bin corresponds to only one attribute, each attribute can contain several state measurements. Thus, each bin can have more than one item. We organise these items into seventeen disjoint non-negative integer intervals so that each bin *B*_*i *_contains the items matching integers in the *i*th interval. Table 4 shows the specified intervals, and the details can be found in [18]. We specify item constraint *IC*_i _in the closed interpretation [see Additional file 1]. Suppose *BV*_i _represents a bin variable.

*IC*_*i*_(*BV*_1_,..., *BV*_*k*_) = *N*_*i*_, 0 ≤ *k *≤ *k*_max _    (3)

*k*_max _is the upper bound of the size of itemsets that users want to find. We specify *N*_i _as the number of itemsets that satisfies constraint *IC*_i _with supports ≥ θ_i_(*BV*_1_, ..., *BV*_k_), where

θi(BV1,...,BVk)={0.044ifλk−1×S(BV1)×⋯×S(BVk)≤0.0441ifλk−1×S(BV1)×⋯×S(BVk)>1λk−1×S(BV1)×⋯×S(BVk)otherwise     (4)
 MathType@MTEF@5@5@+=feaafiart1ev1aaatCvAUfKttLearuWrP9MDH5MBPbIqV92AaeXatLxBI9gBaebbnrfifHhDYfgasaacH8akY=wiFfYdH8Gipec8Eeeu0xXdbba9frFj0=OqFfea0dXdd9vqai=hGuQ8kuc9pgc9s8qqaq=dirpe0xb9q8qiLsFr0=vr0=vr0dc8meaabaqaciaacaGaaeqabaqabeGadaaakeaaiiGacqWF4oqCdaWgaaWcbaGaeeyAaKgabeaakiabcIcaOiabdkeacjabdAfawnaaBaaaleaacqaIXaqmaeqaaOGaeiilaWIaeiOla4IaeiOla4IaeiOla4IaeiilaWIaemOqaiKaemOvay1aaSbaaSqaaiabbUgaRbqabaGccqGGPaqkcqGH9aqpdaGabeqaauaabaqadiaaaeaacqaIWaamcqGGUaGlcqaIWaamcqaI0aancqaI0aanaeaacqWGPbqAcqWGMbGzcqWF7oaBdaahaaWcbeqaaiabdUgaRjabgkHiTiabigdaXaaakiabgEna0kabdofatjabcIcaOiabdkeacjabdAfawnaaBaaaleaacqaIXaqmaeqaaOGaeiykaKIaey41aqRaeS47IWKaey41aqRaem4uamLaeiikaGIaemOqaiKaemOvay1aaSbaaSqaaiabdUgaRbqabaGccqGGPaqkcqGHKjYOcqaIWaamcqGGUaGlcqaIWaamcqaI0aancqaI0aanaeaacqaIXaqmaeaacqWGPbqAcqWGMbGzcqWF7oaBdaahaaWcbeqaaiabdUgaRjabgkHiTiabigdaXaaakiabgEna0kabdofatjabcIcaOiabdkeacjabdAfawnaaBaaaleaacqaIXaqmaeqaaOGaeiykaKIaey41aqRaeS47IWKaey41aqRaem4uamLaeiikaGIaemOqaiKaemOvay1aaSbaaSqaaiabdUgaRbqabaGccqGGPaqkcqGH+aGpcqaIXaqmaeaacqWF7oaBdaahaaWcbeqaaiabdUgaRjabgkHiTiabigdaXaaakiabgEna0kabdofatjabcIcaOiabdkeacjabdAfawnaaBaaaleaacqaIXaqmaeqaaOGaeiykaKIaey41aqRaeS47IWKaey41aqRaem4uamLaeiikaGIaemOqaiKaemOvay1aaSbaaSqaaiabdUgaRbqabaGccqGGPaqkaeaacqWGVbWBcqWG0baDcqWGObaAcqWGLbqzcqWGYbGCcqWG3bWDcqWGPbqAcqWGZbWCcqWGLbqzaaaacaGL7baacaWLjaGaaCzcamaabmGabaGaeGinaqdacaGLOaGaayzkaaaaaa@B227@

where *S*(*BV*_i_) is the smallest support of the items in the bin *BV*_i_. λ is an integer larger than 1 and used to slow down the decrease of *S*(*BV*_1_) × ... × *S*(*BV*_k_) in case of large *k *[16].

### Rule generation

Unlike traditional methods, our approach starts with pruning items of low occurrence, rather than generating *frequent itemsets *directly. *FP-tree *algorithm is implemented on the obtained initial items to generate initial interesting itemsets. The process is repeated until all initial interesting itemsets are extracted. The procedure of finding association rules consists of four phases:

1. Generate *initial interesting itemsets *from *initial items*.

2. Sort out the itemsets containing no filtering symbols.

3. Set up bin *B*_j _and item constraint *IC*_*i*_.

4. Identify association rules using *IC*_*i*_.

The value of *N*_i _is determined by formula (3) and (4). We assign λ with 5 like [12]. *k*_max _= 9 is set using the principle of rule generation in section 3.3 because the itemsets containing more than 9 items are not interesting according to the specified maximum item constraints *IC*(*B*_9_, *B*_10_, *B*_11_, *B*_12_, *B*_13_, *B*_14_, *B*_15_, *B*_16_, *B*_17_). There are 47 item constraints specified in a file for the itemsets in the closed interpretation such as *IC*(*B*_12_, *B*_13_, *B*_14_, *B*_15_, *B*_16_, *B*_17_) and *IC*(*B*_1_, *B*_4_). The constraints are classified into two categories:

1. Contain all items of stimulus factors at least; and

2. Contain only items of AMPK subunits.

They enumerate the items in a bin in the closed interpretation, on the basis that the user is interested in only the items with respect to specification, rather than all possible combinations of items.

Based on *Definition 2*, the obtained itemsets, due to item constraints, need to be sorted in light of their supports in descending order, by which we can generate the *top-N*_i _interesting itemsets for each corresponding bin *IC*_i_. Therefore, the mining will focus on finding out association rules on the basis of these *top-N *interesting itemsets. Table 5 shows the results of *initial interesting itemsets*, *itemsets without filtering symbols*, *sorted itemsets in bins in the closed interpretation *and *top-N interesting itemsets in bins*. From the observation, the search space is greatly reduced as a result of the use of filtering symbols and item constraints.

**Figure 3:**
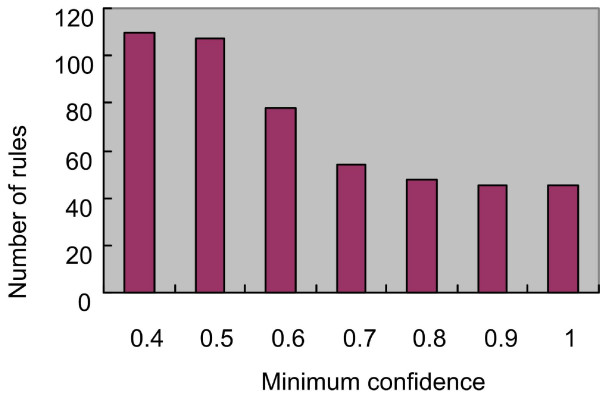
The frequent patterns for AMPK regulation data.

The *top*-*N *interesting itemsets are then used to identify frequent patterns based on the defined criteria in subsection 'Discovering Association Rules', by which some uninteresting rules are pruned. We vary the minimum confidence starting from 0.4 to 1 by increasing 0.1 each time. Figure 3 shows the result of frequent patterns. We observe that there is no sharp drop in rule output when setting the minimum confidence from 0.7 to 1 in comparison with 0.6. Therefore, we select the results by 0.7 in contrast to the results by 0.6 in the following analysis. There are 74 and 51 association rules by minimum confidence 0.6 and 0.7, respectively. From these rules, we can find many potential correlations between itemsets. The rules by 0.7 are classified into the form of Rule1 (40 rules) and the form of Rule2 (11 rules) in terms of the definition in subsection 'Discovering Association Rules'. Nevertheless, the rules need to be pruned because some of them can overlap with each other. Suppose *R*_i_: *A*_i _→ *B*_i _and *R*_j_: *A*_j _→ *B*_j _are two rules. The pruning complies with the principles below:

- *Delete R*_j_, if *A*_i _= *A*_j _and *B*_i _⊇ *B*_j_

- *Replace R*_i _and *R*_j _with *A*_i _∨ *A*_j _→ *B*_i_, if *B*_i _= *B*_j_, *A*_i _*A*_j _and *A*_j _*A*_i_

Finally, we obtain 32 rules after pruning. For example, 29 → 32 are removed due to 29 → 6 32; 14 → 1 and 13 → 1 are replaced by 14 ∨ 13 → 1 where 1, 6, 13, 14, 29 and 32 denote α_1a_↓, α_1e_|, α_2a_↓, α_2a_|, β_2e_| and γ_2e_|, respectively. For convenience, we reconvert the integers into readable symbols in section 'Methods'. Table 6 shows some selected association rules mined from the AMPK regulation data set, in which *t*, *I*, *T*, *ng*, *lg*, *hg*, nc, *ut *and *nd *represent *time*, *intensity*, *trained*, *normal glycogen*, *low glycogen*, *high glycogen*, *untrained *and *normal diabetes*, respectively.

### Performance comparison and feature discovery

Our miner (eFP) extends the FPgrowth* method [19] to identify the potential correlation from AMPK regulation. Table 7 presents a performance comparison between our miner and algorithms FPgrowth [13], dEclat [20] and MAFIA [21]. In the comparison, we use a synthetic sparse dataset *T40I10D100K * and a dense dataset *connect-4 *.

In the FI (frequent itemset) mining, the comparison shows that eFP outperforms FPgrowth, dEclat and MAFLA algorithms, and can still run for a small minimum support. It is observed that both dElcat and MAFIA suffer from high memory consumption and low speed when a small minimum support is used. eFP has almost the same running time as FPgrowth in case of the dense dataset or very low levels minimum support, but shows a speedup when a sparser dataset is applied. eFP uses the compact FPtree [19], it not only spends less time on constructing and traversing the trees, than the time on TID-array intersections (dEclat) and bitvector *and *operations (MAFLA), but also needs less main memory space for storing FP-trees than that for storing diffsets or bitvectors. Thus eFP runs faster than the other three algorithms, and it performs well, even when giving very small minimum support.

In addition, our framework includes some novel features, by which to efficiently identify the association rules of interest. Preprocessing of experiment data is used to convert the qualitative data into quantitative data (non-negative integers). Particularly, filtering symbols are used to assist in pruning the untested items. They facilitate the identification of frequent itemsets. The constraint-based mining technique, namely top-*N *interesting itemsets, provides a flexible way for users to specify a set of constraints and allow them to search and control the interesting frequent patterns. The experiments discover a number of interesting frequent patterns that make sense biologically. They not only demonstrate former experiments but also predict some potential results that were unknown previously. These can benefit to the understanding of the signalling pathway of AMPK in regulating metabolism, and disease diagnosis and treatment.

### Interpretation

AMP-activated protein kinase (AMPK) is a protein kinase which is ubiquitously expressed and functioned as a stress sensor of the cellular energy status. A functional AMPK exists as a heterotrimetric complex comprising one catalytic subunit α and two regulatory subunits β and γ. In mammals, each subunit is encoded by multiple genes (α_1_, α_2_, β_1_, β_2_, γ_1_, γ_2_, γ_3_) [22]. AMPK activity is regulated not only by cellular AMP/ATP ratio, but also by upstream kinases. The skeletal muscle is one of most energy turnover tissue in mammals. AMPK was found to play an important role in the regulation of muscle metabolism. We have selected 45 experiments [18] in relevant bio/medical literatures in Medline by searching the NCBI using keywords "*AMPK and human skeletal muscle*" (103 papers) and "*AMPK and endurance training*" (16 papers), respectively.

We try to find out potential relationships between the AMPK isoform expression and activity, and association between subunits. As mentioned above, this relationship is critical to assist biologists in further predicting AMPK pathways. These are usually hidden in experimental data and these pathways are not easy to identify by traditional statistics. From the experimental results in this study, we have selected 12 association rules to describe AMPK regulation in skeletal muscle, shown in Table 6. For example, as for rule 1 in Table 6, the items from the rule are mentioned together in 27% of the relevant experiments, but only 2% or 0% for those items in other rules, such as {*ng t*^- ^*nc nd I*^++ ^*T*} and {α_1a_↓} (2%), {*ng t*^++ ^*nc nd I*^++ ^*T*} and {α_1a_↓} (0%), {*ng t*^+ ^*nc nd I*^++ ^*T*} and {α_1a_|} (2%), {*ng t*^+ ^*nc nd I*^+ ^*ut*} and {α_1a_|} (0%), {*ng t*^+ ^*nc nd I*^++ ^*ut*} and {α_1a_↑} (2%) and {*ng t*^++ ^*nc nd I*^++ ^*ut*} and {α_1a_↑} (0%). In other words, the items in rule 1 occur much more frequently than those from other rules under the same indexes. The remaining rules can be explained in the same way. These can be secondary evidence to support the fact that our chosen rules are more important than the other ones.

The rules by 0.7 (minimum confidence) in Table 6 are a subset of the originally obtained rules [see Additional file 2]. They not only show that in most of the experiments where stimuli were imposed the isoforms of AMPK can be activated, but also represent that specific correlations between the states of isoforms exist. For example, rule 4 in Table 6 states that in most of the cases where α_1a _was highly expressed, α_2a _was highly expressed too. The rest of the rules in Table 6 can be interpreted in a similar manner. In particular, the rules that are not previously known will be highlighted.

Looking at Table 6 and the supplementary rules, it's seen that the expression of α, β and γ varies from diverse exercise stimuli. In particular, skeletal muscle expresses predominantly the α_2 _and β_2 _subunits. Referring to the rules by 0.6, we see β_1_, β_2_, γ_1 _and γ_3 _except γ_2e _are also activated (interacting with α) in response to endurance training. This is due to β and γ that are two regulatory subunits, they are activated only when they are associated with α subunit. However, these rules have a slightly lower confidence than the cutoff threshold (70%), which would help explain their absence in the rules by 0.7. Synthetically, these factors/subunits/isoforms α_1a_, α_2a_, α_1p_, α_2p_, α_1e_, α_2e_, β_1e_, β_2e_, γ_1e _and γ_3e _are actively involved in regulating the metabolism in response to energy demand and supply in skeletal muscle. We seek more experiments to ascertain the characteristic of γ_2e _(in white muscle). In addition, we see a number of rules that state the latent correlation between the isoforms of AMPK. Most of them are new and make sense biologically. Furthermore, we can predict some latent associations that are selected for biologists to test in future experiments.

From our observations, most of the found rules except rule 2, 3, 4, 5 were unknown previously. They are important due to the identification of innovative correlations between AMPK subunits, which imply meaningful information for understanding their association and regulation in signalling pathways. Recent experimental results also demonstrate this [4,23,24]. We also view rule 1 as a type of specific rule using disjunctive normal form in the antecedent. This rule is able to integrate the knowledge from three sub-rules and lead to new knowledge using comparison amongst and between them. The details can be seen below.

Looking at rule 1 in Table 6, the activation of α_1_-AMPK usually has no change if the exercise intensity is not high enough and the duration is not extra long. Regardless of the status of training, glycogen, diabetes and nicotinic acid, α_1 _activity remains unchanged. Although some experiments suggested that AMPK can be substantially activated during maximal sprint-type exercise in humans [25-27]. Unfortunately, this rule cannot be generated at this stage due to insufficient experimental support. Rule 1 is a novel point since we only have seen α_1 _activity changes in very few experiments. In addition, it is innovative because the disjunction normal form of antecedents may simultaneously integrate information from multiple experiments. Thus, we determine that α_1 _activity cannot be significantly affected by the status of training, glycogen, diabetes and nicotinic acid because α_2 _instead of α_1 _predominantly localized in skeletal muscle. Such comparison cannot be achieved through traditionally isolated experiments. This can be tested in future experiments and aids in understanding the properties of α_1_. Thus, the regulation of AMPK in skeletal muscle is probably relied on α_2 _rather than α_1_. Furthermore, AMPK may be a critical mediator or exercise-induced changes in glucose uptake and fatty acid oxidation in human skeletal muscle, and the AMPK α_2_-containing heterotrimer is possible to be the predominant complex responsible for the regulation in both healthy and diabetic subjects.

Looking at rules 2, 3 and 4 in Table 6, α_2a _is expressed in skeletal muscles from untrained to trained individuals. Nevertheless, α_2a _is only highly expressed in skeletal muscle from well-trained individuals in both moderate-intensity and high-intensity training. Importantly, α_2 _activity is probably activated more easily in skeletal muscle from untrained individuals than well-trained individuals at the same relative intensity. These associations are in accordance with that of [1,28-30]; α_1a _is usually unchanged below high-intensity training, which is in agreement with the results of [29,31]. A recent study also supports these rules [32]. These findings reveal that α_2 _and α_1_, may play different physiological roles in mediating metabolic events in skeletal muscle. Actually, AMPK α_2 _is predominantly localized in skeletal muscle, heart and liver, whereas α_1 _is widely distributed. In addition, the acute activation of α_2_-AMPK during exercise may result in not only a significant increased glucose disposal in muscle but also decreased malonyl-CoA contents, which might ameliorate insulin resistance and improve glycemia [1]. These may explain the above rules. Indeed, AMPK signalling was reduced by either overexpressing a kinase-dead α_2_-AMPK construct or knocking out the catalytic α_2_-isoform [33,34]. Although an increase in α_2 _activity of subjects with type 2 diabetes was found during acute exercise, accompanying a significant decrease in blood glucose concentrations [1], we need more results to establish this rule.

Looking at rule 5, α-AMPK phosphorylation on the α_1 _and α_2 _are increased in skeletal muscle from well-trained individuals with high-intensity and moderate duration training. This is because the phosphorylation of α_1 _and α_2 _is more relative to cell regulation by environment stress. In contrast, the expression of α_2 _tends to be gene regulation. This suggests that AMPK activity or upstream kinase(s) is being regulated by training, which is in agreement with the results of [3,35,36]. A newly published literature also defends this rule [37].

Looking at rules 6 and 7, both AMPK α_1 _and α_2 _expressed in skeletal muscle. There is no clear evidence that α_2 _activity is leaded by α_1 _since they are actually independent with each other. In [33], α_1 _protein expression was increased 2–3-fold in α_2 _knockout mice, while α_2 _protein level in α_1 _knockout mice is comparable with that observed in WT mice. Since α_1 _activity is usually unchanged while α_2 _activity is easily increased under the same exercises. α_1 _activity might change only under higher intensity exercises. In contrast, α_2 _activity will certainly go up under the same condition (higher intensity). That is why we get Rule 6.

Looking at rule 8, in comparison with α_2_, α_1 _activity mostly remains unchanged, notwithstanding the high increase of phosphorylation of α_1 _because α_2 _phosphorylation should be higher and contributed to higher α_2 _activity after exercise. Actually, the phosphorylation of α is usually presented as a ratio of phosphorylated α divided by total α. Although α_1 _phosphorylation may slightly increases, this cannot cause the increase of α_1 _activity. As to our knowledge, no literature in Medline previously points out the relationship between phosphorylation and expression of α_2_. The experimental results in [38] also match our computational discovery.

Rule 9 in Table 6 shows that α activity is co-expressed with expression of β or γ. α_2 _activity rather than α_1 _activity appears to correlate with expression of β and γ subunits. Co-expression of α subunits with β or γ subunits modestly increases kinase activity accompanied by the formation of α/β or α/γ heterodimers. In addition to binding of each noncatalytic subunit to the α subunit, β and γ subunits bind to each other, possibly resulting in a more stable heterotrimeric complex [39]. The increase in kinase activity associated with expression of this heterotrimer is due both to an increase in enzyme-specific activity (units/enzyme mass) and to an apparent enhanced α subunit expression. Co-expression of the noncatalytic β or γ subunits is required for optimal activity of the α catalytic subunits. This may explain the possible and/or necessary co-expression of α with β and γ subunits. In the same way, we can explain Rule 10, which represents that if the expression of γ_1 _and γ_3 _are significantly increased, the expression of β_1_, β_2 _and γ_2 _will increase too.

Rule 11 does not make sense in biology because there is no relationship between the phosphorylation of α_1 _and α_2_. Rule 12 shows the protein expression of α_1 _is co-expressed with the expression of β_2. _This may imply that the tight relation between γ_3 _and α_2_, γ_3 _and β_2_, γ_1 _and both α_1 _and α_2_, and γ_1 _and β_2_. The new experimental results in [23,24] defend these rules. There are 12 theoretically possible AMPK heterotrimetric complexes. But in human skeletal muscle, there are only three detectable combinations exist α_2_β_2_γ_1_>>α_2_β_2_γ_3 _= α_1_β_2_γ_1 _[24]. Our results predict that there maybe α_1_β_1_γ_1 _complex in human skeletal muscle as well.

Furthermore, we can predict some latent associations from the derived association rules. For example, if the training is not of high-intensity, we can predict deductively α_1 _activity in terms of rule 1 in Table 6. Besides, the newly found association rules can be used in the design of future experiments. For example, α_2a _was not highly activated at rest status. If we want to activate it, we can regulate the intensity or duration of exercise as indicated in rules 2, 3 and 4 in Table 6. Therefore, the identified association rules may play important roles in three aspects:

- demonstrating former experiments via matching more experimental results;

- predicting potential results based on existing conditions; and

- guiding the design of future experiments.

From our experiments, we demonstrated that association rules can not only discover important biological patterns but also be used to reduce the cost of labor, resources and other associated activities. Experiments can be conducted based on the derived association rules, thereby reducing the number of experiments. For example, if α_2 _activity is increased with exercise in one experiment, we can predict that α_1a _possibly has no change under the same condition based on rules 1 and 2 in Table 6. Similarly, if α_1a _is highly increased with high intensity training, we can predict that α_2a _is possibly highly increased either in light of rule 6. Therefore, it can save the experimental time by avoiding extra (unnecessary) stimuli. For example, rule 4 in Table 6 implies that *high-intensity *training can result in high expression of α_2a_. If we want to observe that α_2a _is highly expressed in experiments, we can purposefully handle *high-intensity *stimuli rather than *intense-intensity*. Consequently, the rules are beneficial to understand the signalling pathway of AMPK in regulating metabolism and its potential benefits to disease treatment.

## Discussion

In this study, we have proposed a framework by which to identify association rules of interest, either between the state of isoforms of α, β and γ subunits of AMPK, or between stimulus factors and the state of isoforms, from AMPK regulation data. In particular, the item constraints are applied in the closed interpretation to the itemset generation. We have shown how to specify a threshold in terms of the amount of results instead of a fixed threshold for all itemsets.

Our approaches start with collecting hidden data from publications in Medline. The collected experimental data is qualitative and does not correspond with the data mining softwares [19]. Besides, we have shown that untested items in some experiments may result in many unrelated or even inaccurate rules. If the untested items are not pruned, it seemed to cause many inaccurate results, and the implementation of software became less efficient when identifying frequent patterns [8]. Consequently, we marked the untested items using the filtering symbols, which facilitate the pruning of frequent itemsets and avoid the generation of irrelevant frequent itemsets. To meet the criteria of softwares in [19], it is needed to conduct data preprocessing and convert the qualitative items into the form of quantitative items (non-negative integers), which benefit to the execution of software.

The traditional association rule mining typically requires a user to specify a minimum support threshold for the generation of all itemsets. It has been argued that without specific knowledge, it is difficult for users to obtain an optimal support threshold. It was showed that a better way is to identify top-*N *interesting itemsets, instead of specifying a fixed threshold value for all itemsets of all sizes like [12,13,16]. However, a major problem is that not all top-*N *itemsets are interesting. Some correlations might not make sense biologically at all. The item constraints in the closed interpretation are applied to the itemset generation. For example, in AMPK regulation, the relationships between the activity of *subunit isoforms *and *stimulus factors *such as intensity and duration are interesting but the relationships between *stimulus factors *are not. It provides a flexible way for users to specify a set of constraints and allow them to search and control the interesting frequent patterns.

In our selection of association rules, we adopt 0.7 as the minimum confidence because there is no sharp drop in rule output in contrast with 0.6. Although this helps us focus on the interpretation of significant rules, some rules that have a close confidence to 0.7 might be ignored. Extending the interpretation to those less important rules is therefore desirable, but it would require more computational resources and collaboration with biologists.

Bayesian network, a graphic model, has been widely used to identify metabolic pathways and construct genetic networks due to its statistical significance and inherited advantage in handling the information with uncertainty. Nevertheless, as an assumption-driven method, it relies on the quality and extent of the prior beliefs and is only as useful as this prior knowledge is reliable. However, the hidden and insufficient AMPK regulation data in the publications of Medline prevents us from obtaining reliable prior knowledge. Furthermore, some potential AMPK pathways are undetermined and might be ignored from the assumption. Considering the above difficulties, the authors turn to data mining, a data-driven method, in this study.

We have focused our attention here on human skeletal muscle. Also, our methods are eligible for other organisms, such as mouse, where the experimental indexes or criteria may be quite different. A modification with respect to data preprocessing may be adopted according to the criteria. Another interesting question is whether our methods can be used to explore the AMPK regulation on other tissues and organs, such as adipose tissue, heart and liver [4]. Intuitively, it is necessary to classify the data into different groups because different compositions may play a similar role on different tissues or organs. On the other hand, studying the potential metabolic pathways between AMPK regulations on different tissues or organs should be useful in disease prediction, diagnosis and treatment. We plan to seek answers for these questions in our future work. The results can enable biologists to understand AMPK pathways and extend it to the other kinases to form a full kinase interaction mapping.

## Conclusion

AMPK has emerged as an important energy sensor in the regulation of cell metabolism. Recent experiments reveal that physical exercises are closely linked with AMPK activation in skeletal muscle. This paper proposes a framework by which to identify association rules of interest from the published experimental data.

Unlike the conventional methods, the measurement of items from AMPK regulation data is taken into account. In addition, the items that have low occurrence in existing experiments are pruned prior to mining. Furthermore, we apply item constraints in the closed interpretation to the itemset generation so that a threshold is specified on the amount of results rather than a fixed threshold, thereby reducing the search space vastly.

Our framework was demonstrated by mining realistic AMPK regulation data set with respect to skeletal muscle. Many of the found rules make sense biologically, others suggesting new hypotheses that may warrant further investigation. Particularly, some of them were unknown previously. Moreover, they help us understand the characteristics of AMPK and relevant disease treatment. It is thus promising in the interpretation of AMPK regulation data.

## Authors' contributions

QFC conceived of and carried out the AMPK regulation studies, applied the data mining method to AMPK data sets, interpreted biologically the experimental results and wrote the manuscript. YPPC supervised the project and suggested ways of improving the study, and participated in writing the manuscript. Both authors read and approved the final version of the manuscript.

## Supplementary Material

Additional file 1 An implementation of the FP-tree-based algorithm. The files consist of the AMPK regulation data with respect to the human skeletal muscle, and the applied item constraints in this study. Click here for file

Additional file 2 Our results from mining AMPK regulation data set regarding human skeletal muscle. The results provided present the interesting frequent patterns with respect to the AMPK pathways. Click here for file

## Figures and Tables

**Table 1:** An Example of Experimental Database

EID	Items						
	
	α_1_			β		φ	φ
*E*_1_	α_1a_↓	-	α_1p_↑	-	-	φ^+^	φ^-^
*E*_2_	α_1a_↓	α_1e_|	α_1p_|	β_1e_|	β_2e_|	φ^+^	φ^-^
*E*_3_	α_1a_↓	α_1e_|	-	β_1e_|	-	φ^+^	φ^+^
*E*_4_	α_1a_|	α_1e_↓	α_1p_|	β_1e_|	β_2e_|	φ^++^	φ^+^

**Table 2:** A Converted Experiment Dataset

**EID**	**Items**						
	
	α_1_				β	φ	φ
***E*_1_**	1	8	11	27	30	91	100
***E*_2_**	1	6	10	26	29	91	100
***E*_3_**	1	6	12	26	30	91	101
***E*_4_**	2	5	10	26	29	92	101

**Table 3:** Activity and expression of α_1a _and α_1e _of AMPK in skeletal muscle

**α_1a_**	**α_1e_**	***Training***	***Glycogen***	***Diabetes***	***N***	***I***	***D***
2	5	50	60	70	80	92	101
1	5	51	60	70	80	90	103
1	6	51	60	70	80	91	100
1	6	51	60	70	80	91	101

**Table 4:** The bins and corresponding interval

***B*_i_**	**Attibute**	**Interval**	***B*_i_**	**Attibute**	**Interval**
***B*_1_**	α_1a_	[1, 4]	***B*_10_**	γ_2e_	[34,37]
***B*_2_**	α_1e_	[5, 8]	***B*_11_**	γ_3e_	[38, 40]
***B*_3_**	α_1a_	[9 12]	***B*_12_**	training	[50, 51]
***B*_4_**	α_1a_	[13, 16]	***B*_13_**	glycogen	[60, 62]
***B*_5_**	α_1e_	[17, 20]	***B*_14_**	diabetes	[70, 71]
***B*_6_**	α_2p_	[21, 24]	***B*_15_**	nicotinic acid	[80, 81]
***B*_7_**	α_1e_	[25, 27]	***B*_16_**	intensity	[90, 93]
***B*_8_**	α_2e_	[28, 30]	***B*_17_**	duration	[100, 103]
***B*_9_**	γ_1e_	[31, 33]			

**Table 5:** The result of itemset generation

**Itemset**	**Number**
*Initial interesting itemset*	964519
*Itemset without filtering symbols*	14985
*Sorted itemset in bins*	97
*Top-N interesting itemset in bins*	97

**Table 6:** Selected association rules from AMPK regulation data set

**Association rule**	
1	{*t*^+ ^*ng I*^+ ^*nc ut nd*} ∨ {*hg t*^+ ^*nc nd I*^+ ^*T*} ∨ {*t*^++ ^*nc I*^+ ^*ut ng nd*} → {α_1a_↓}
2	{*lg T nd nc I*^+ ^*t*^+^} → {α_1a_↓α_2a_|}
3	{*I*^++ ^*T t*^+ ^*nd nc ng*} → {α_2a_|}
4	{*I*^++ ^*t*^- ^*ut nc nd ng*} → {α_2a_↑}
5	{*I*^++ ^*t*^+ ^*nc nd ng T*} → {α_2p_|α_1p_|}
6	{α_1a_↑} → {α_2a_↑}
7	{α_1e_|} → {α_2e_|}
8	{α_1p_↑} → {α_1a_↓α_2a_↑α_2p_↑}
9	{β_2e_|β_1e_|} ∨ {γ_2e_↑γ_1e_|γ_3e_|} → {α_2a_↑α_1a_↓}
10	{γ_1e_|γ_3e_|} → {γ_2e_↑β_2e_|β_1e_|}
11	{α_1p_|} → {α_2p_|}
12	{α_1e_|α_2e_↓} → {β_1e_↓β_2e_|}

**Table 7:** Performance comparison in mining FI (frequent itemsets)

**Miner**	**Data set**	**Minimum support (%)**	**CPU Time(s)**
***eFP***	*T40I10D100K*	0.05 ≤ *minsupp *≤ 2	[4.5, 390]
***FPgrowth***	*T40I10D100K*	0.05 ≤ *minsupp *≤ 2	[9.5, 390]
***dEclat***	*T40I10D100K*	0.25 ≤ *minsupp *≤ 2	[7.9, 400]
***MAFIA***	*T40I10D100K*	0.75 ≤ *minsupp *≤ 2	[60, 5936]
***eFP***	*connect-4*	20 ≤ *minsupp *≤ 90	[0.04, 740]
***FPgrowth***	*connect-4*	20 ≤ *minsupp *≤ 90	[0.04, 740]
***dEclat***	*connect-4*	20 ≤ *minsupp *≤ 60	[1, 97]
***MAFIA***	*connect-4*	20 ≤ *minsupp *≤ 80	[8.1, 593]
