# Supplementary material for: Mining frequent patterns for AMP-activated protein kinase regulation on skeletal muscle
Source: BMC Bioinformatics. 2006 Aug 30;7:394. doi: 10.1186/1471-2105-7-394 (PMC1574354; doi:10.1186/1471-2105-7-394)
Supplement: Additional file 1 — An implementation of the FP-tree-based algorithm. The files consist of the AMPK regulation data with respect to the human skeletal muscle, and the applied item constraints in this study. [file 1471-2105-7-394-S1.zip › executionreademe.rtf]

The fpbin.zip consists of ampk.dat, ampktab.xls, constraint.dat, filterbin.txt, resultf2a.dat and fim.tar.

1 ampktab.xls is the original data that includes remarks such as the notations, publication date and authors

2 ampk.dat is the dataset extracted from ampktab.xls.

3 constraint.dat is used to specified the item constraints for the item generation

4 fim.tar is software for mining frequent itemsets. The details about its use can be found by http://www.cs.concordia.ca/db/dbdm/dm.html.

5 filterbin.txt is the source code by Perl.

6 resultf2a.dat stores the frequent itemsets after filtering the untested items

How to use the source code?

1 Extract all frequent itemsets from ampk.dat using software in fim.tar.

2 Save the results into result2a.dat that includes the initial frequent itemsets

3 Execute of the program filter.bin

4 The program is executable with two parameters: 

  *  The path of the dataset-file,
  *  The minimal confidence threshold, which is a non-negative integer.

5  After execution of the program, the output file contains a list of association rules.
