# Supplementary material for: Mining frequent patterns for AMP-activated protein kinase regulation on skeletal muscle
Source: BMC Bioinformatics. 2006 Aug 30;7:394. doi: 10.1186/1471-2105-7-394 (PMC1574354; doi:10.1186/1471-2105-7-394)
Supplement: Additional file 2 — Our results from mining AMPK regulation data set regarding human skeletal muscle. The results provided present the interesting frequent patterns with respect to the AMPK pathways. [file 1471-2105-7-394-S2.zip › resultreademe.rtf]

The skele_mus.zip consists of eight files, including asso_rule0.4.dat, asso_rule0.5.dat, asso_rule0.6.dat, 
asso_rule0.7.dat, asso_rule0.8.dat, asso_rule0.9.dat, asso_rule1.dat and asso_rule0.7prune.txt.

1 asso_rule0.4.dat, asso_rule0.5.dat, asso_rule0.6.dat, asso_rule0.7.dat, asso_rule0.8.dat, asso_rule0.9.dat, and asso_rule1.dat 
represent the derived association rules by minimal confidence from 0.4 to 1, respectively.

2 . asso_rule0.7prune.txt is the compression form of asso_rule0.7.dat by integrating relevant rules together.
