# Supplementary material for: Mining frequent patterns for AMP-activated protein kinase regulation on skeletal muscle
Source: BMC Bioinformatics. 2006 Aug 30;7:394. doi: 10.1186/1471-2105-7-394 (PMC1574354; doi:10.1186/1471-2105-7-394)
Supplement: Additional file 2 — Our results from mining AMPK regulation data set regarding human skeletal muscle. The results provided present the interesting frequent patterns with respect to the AMPK pathways. [file 1471-2105-7-394-S2.zip › asso_rule0.7prune.txt]

 3 -->  15 is a rule of interest
 6 -->  18 is a rule of interest
 5 -->  10 22 17 is a rule of interest
 10 -->  22 is a rule of interest
 11 -->  1 15 23 is a rule of interest
 13  Ú 14 -->  1 is a rule of interest
 18 -->  6 is a rule of interest
 22 -->  10 is a rule of interest
 29 -->  6 32 is a rule of interest
 32 -->  29 6 is a rule of interest
 36 -->  29 32 26 39 is a rule of interest
 36 -->  32 39 15 1 is a rule of interest
 38 -->  29 32 is a rule of interest
 39 -->  36 29 32 26 is a rule of interest
 39 -->  36 32 15 1 is a rule of interest
 5 17 -->  10 22 is a rule of interest
 6 17 -->  25 29 is a rule of interest
 11 23 -->  1 15 is a rule of interest
 29 26  Ú  36 32 39 -->  15 1 is a rule of interest
 25 29 -->  6 17 is a rule of interest
 29 26 -->  36 32 39 is a rule of interest
 29 32 -->  6 is a rule of interest
 38 32 -->  29 is a rule of interest
 32 39 -->  36 29 26 is a rule of interest
 32 39 -->  36 15 1 is a rule of interest
 29 32 39 -->  36 26 is a rule of interest
 36 32 39 -->  29 26 is a rule of interest
 101 60 91 80 51 70  Ú 62 101 80 70 91 50  Ú 102 80 91 51 60 70 -->  1 is a rule of interest
 92 100 51 80 70 60 -->  15 is a rule of interest
 92 50 101 70 80 60 -->  14 is a rule of interest
 61 50 70 80 91 101 -->  1 14 is a rule of interest
 92 101 80 70 60 50 -->  22 10 is a rule of interest
